# A Mobile Phone–Based Support Intervention to Increase Use of Postabortion Family Planning in Cambodia: Cost-Effectiveness Evaluation

**DOI:** 10.2196/16276

**Published:** 2020-02-25

**Authors:** Jeremy Hill, Jourdan McGinn, John Cairns, Caroline Free, Chris Smith

**Affiliations:** 1 Department of Global Health and Development London School of Hygiene and Tropical Medicine London United Kingdom; 2 Department of Health Services Research and Policy London School of Hygiene and Tropical Medicine London United Kingdom; 3 Department of Population Health London School of Hygiene and Tropical Medicine London United Kingdom; 4 Graduate School of Tropical Medicine and Global Health Nagasaki University Nagasaki-shi Japan; 5 Department of Clinical Research London School of Tropical Medicine London United Kingdom

**Keywords:** mHealth, digital health, cost-effectiveness, contraception, postabortion contraception, postabortion family planning, Cambodia

## Abstract

**Background:**

Despite progress over the last decade, there is a continuing unmet need for contraception in Cambodia. Interventions delivered by mobile phone could help increase uptake and continuation of contraception, particularly among hard-to-reach populations, by providing interactive personalized support inexpensively wherever the person is located and whenever needed.

**Objective:**

The objective of this study was to evaluate the cost-effectiveness of mobile phone–based support added to standard postabortion family planning care in Cambodia, according to the results of the MOTIF (MObile Technology for Improved Family planning) trial.

**Methods:**

A model was created to estimate the costs and effects of the intervention versus standard care. We adopted a societal perspective when estimating costs, including direct and indirect costs for users. The incremental cost-effectiveness ratio was calculated for the base case, as well as a deterministic and probabilistic sensitivity analysis, which we compared against a range of likely cost-effectiveness thresholds.

**Results:**

The incremental cost of mobile phone–based support was estimated to be an additional US $8160.49 per 1000 clients, leading to an estimated 518 couple-years of protection (CYPs) gained per 1000 clients and 99 disability-adjusted life-years (DALYs) averted. The incremental cost-effectiveness ratio was US $15.75 per additional CYP and US $82.57 per DALY averted. The model was most sensitive to personnel and mobile service costs. Assuming a range of cost-effectiveness thresholds from US $58 to US $176 for Cambodia, the probability of the intervention being cost-effective ranged from 11% to 95%.

**Conclusions:**

This study demonstrates that the cost-effectiveness of the intervention delivered by mobile phone assessed in the MOTIF trial lies within the estimated range of the cost-effectiveness threshold for Cambodia. When assessing value in interventions to improve the uptake and adherence of family planning services, the use of interactive mobile phone messaging and counselling for women who have had an abortion should be considered as an option by policy makers.

**Trial Registration:**

ClinicalTrials.gov NCT01823861; https://clinicaltrials.gov/ct2/show/NCT01823861

## Introduction

Contraception provides significant benefits for the health of women and children, as well as substantial social and economic benefits [1]. An estimated 225 million women in developing countries had an unmet need for contraception in 2014, and if the need were met, it could avert 52 million unintended pregnancies, 24 million abortions (of which around half are unsafe), 70,000 maternal deaths, and 500,000 newborn deaths per year [2].

In Cambodia, over the last decade, progress has been made in reducing an unmet need for contraception. This has coincided with a reduction in maternal, infant, and under-5 mortality [3]. Nonetheless, there is a continued unmet need for contraception in Cambodia. The 2014 Cambodia Demographic and Health Survey reported that among married women aged 15-49 years who wanted to delay a pregnancy by more than 2 years or have no further children, only 56% were using contraception [3]. There has been a rise in the rate of induced abortions from 21 per 1000 women in 2005 to 28 in 2010, with 26% of women having more than one abortion [4].

Interventions delivered by mobile phone could help increase uptake and continuation of contraception, particularly among hard-to-reach populations [5-9]. Compared with face-to-face interventions, mobile phone–based interventions have the advantage that they can provide interactive personalized support inexpensively wherever the person is located and whenever needed [10]. The use of this technology could be of value to women who have had an abortion, as they may face stigma when seeking services or may find it difficult to make informed decisions about family planning at the time of their abortion.

The MOTIF (MObile Technology for Improved Family planning) trial evaluated an intervention delivered by mobile phone to provide postabortion family planning support to women who received safe abortion at Marie Stopes International Cambodia (MSIC) clinics [11]. This trial compared usage of different family planning services during a period of 12 months after abortion among women who were provided with family planning advice via their mobile phones (six automated interactive voice messages over 3 months with a facilitated link to counsellor phone support via a call center and appointment booking if requested) in addition to standard postabortion family planning care provided in accordance with national guidelines, with usage of family planning services among women receiving standard care alone.

The MOTIF intervention was effective at increasing uptake of long-acting reversible contraceptive methods (subdermal implant and intrauterine device [IUD]), which are associated with lower discontinuation rates compared with those of short-acting hormonal methods [12-14]. Long-acting methods are more cost-effective in comparison with short-term methods [15,16], but little evidence exists on the cost-effectiveness of behavior change interventions aimed at improving the uptake of these methods. We aimed to conduct a cost-effectiveness analysis of the MOTIF trial intervention to address this evidence gap.

## Methods

### Rationale and assumptions

**Figure 1:**
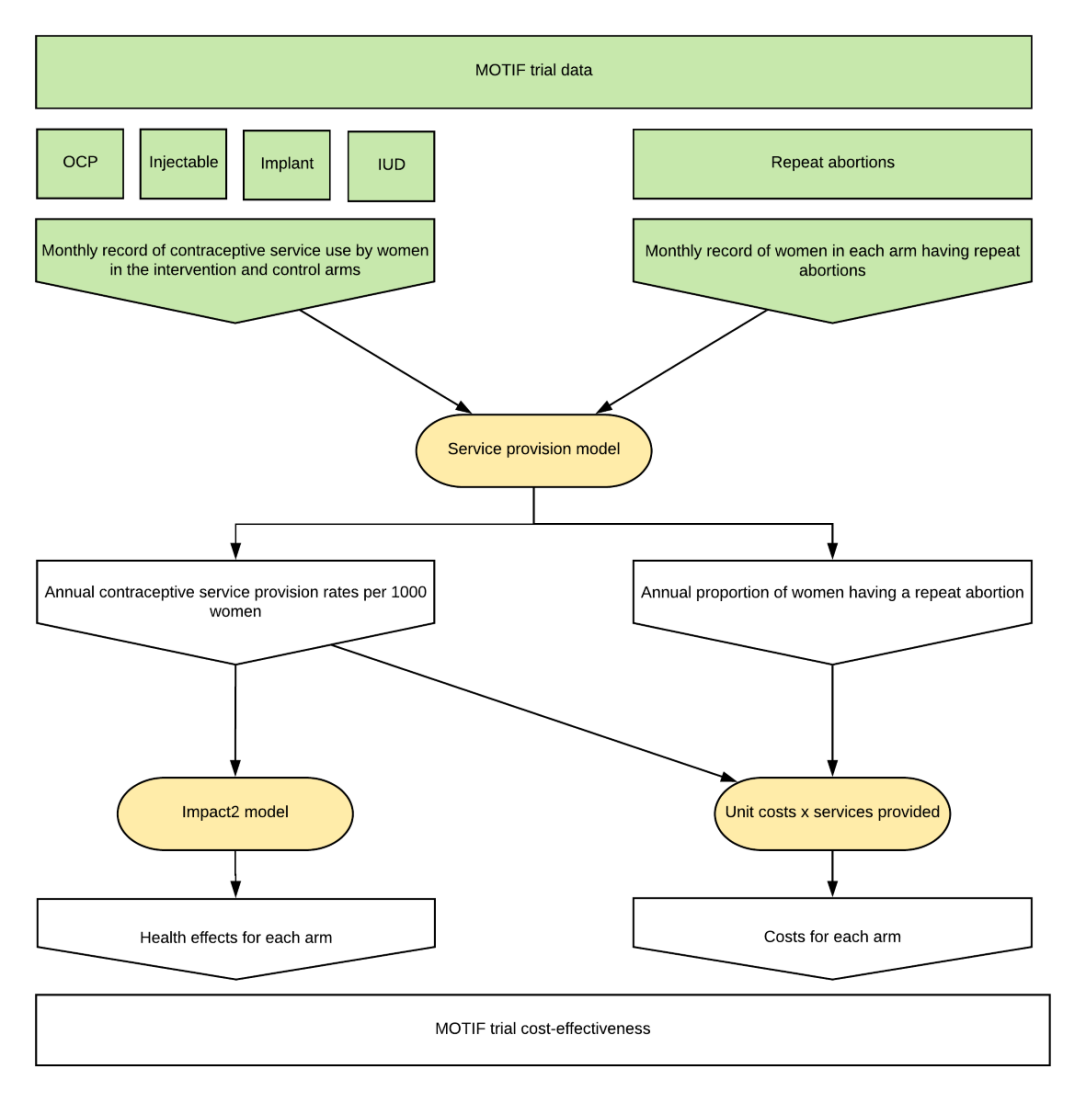
Conceptual framework for the service provision model based on the MOTIF trial. Inputs from the MOTIF trial are shown in green. Models used to derive costs and effects are shown in yellow. IUD: intrauterine device; MOTIF: MObile Technology for Improved Family planning; OCP: oral contraceptive pill.

We conducted a cost-effectiveness evaluation comparing a mobile phone–based intervention in addition to standard postabortion family planning care with standard care alone, using the results of the MOTIF trial. The methods and results of this trial have been previously published [11,14]. In short, standard care included counselling at the clinic, offer of follow-up appointment, and provision of the contact details of an MSIC counselling hotline. Those allocated to the intervention also received six automated interactive voice messages and were provided with phone support from a counsellor depending on their responses to the messages, and optional additional reminder messages were provided to those women who chose to receive oral or injectable contraceptives (a detailed description is provided in Multimedia Appendix 1). The conceptual framework for the cost-effectiveness evaluation is shown in Figure 1. Because postabortion family planning care is delivered over a limited period of time after induced abortion for each individual but the effects of long-acting contraceptive methods may be accrued over the lifespan of the product without further costs being incurred, we chose to model the activities required to deliver postabortion family planning care to a cohort of women along with overhead costs for 1 year only. The time horizon for effects was 10 years, according to the parameters of the Impact2 model as described below.

### Service Provision Model

We constructed a model to simulate total contraceptive and abortion services obtained for a single cohort of 1000 women after abortion in the intervention and control arms, using Excel 2016 (Microsoft Corp, Redmond, Washington). This design was chosen to link the empirical service usage data from the MOTIF trial (monthly services per user) to the Impact2 model (annual services per 1000 users). No discounting was applied to costs, because these were modelled to occur during 1 year.

Monthly service provision parameters were taken from 66% (328/500) of participants remaining in the study at the end of the 12-month follow-up period. Previously published MOTIF findings showed that missing data had a negligible effect on the contraceptive method mix at 12 months [17]. Moreover, some participants in the MOTIF trial used more than one type of contraceptive service (owing to discontinuation or switching). The use of 12-month follow-up parameters allowed us to more accurately reflect this in the overall service provision rates for the simulated cohort of 1000 women in each arm. Model parameters are presented in Table 1. Effects were calculated using contraceptive service parameters only, whereas costs were calculated using both contraceptive service and abortion service parameters. Contraceptive service provision rates and confidence intervals were derived from monthly MOTIF trial data.

**Table 1:** Service provision model parameters.

			Base	Deterministic range (95% CI)		Probabilistic distribution	
**Intervention arm and parameters**							
	**Contraception services^a^**						
		Oral contraceptive pill	2172		2013-2330		Lognormal
		Injectable	558		512-604		Lognormal
		Implant	172		123-220		Lognormal
		IUD^b^	112		72-153		Lognormal
	**Abortion services^a^**						
		Repeat abortion	47		21-91		Beta
**Control arm parameters**							
	**Contraception services^a^**						
		Oral contraceptive pill	3308		3112-3499		Lognormal
		Injectable	325		291-358		Lognormal
		Implant	75		42-109		Lognormal
		IUD	63		32-93		Lognormal
	**Abortion services^a^**						
		Repeat abortion	69		35-120		Beta

^a^Per 1000 participants per year.

^b^IUD: intrauterine device.

### Effects

**Figure 2:**
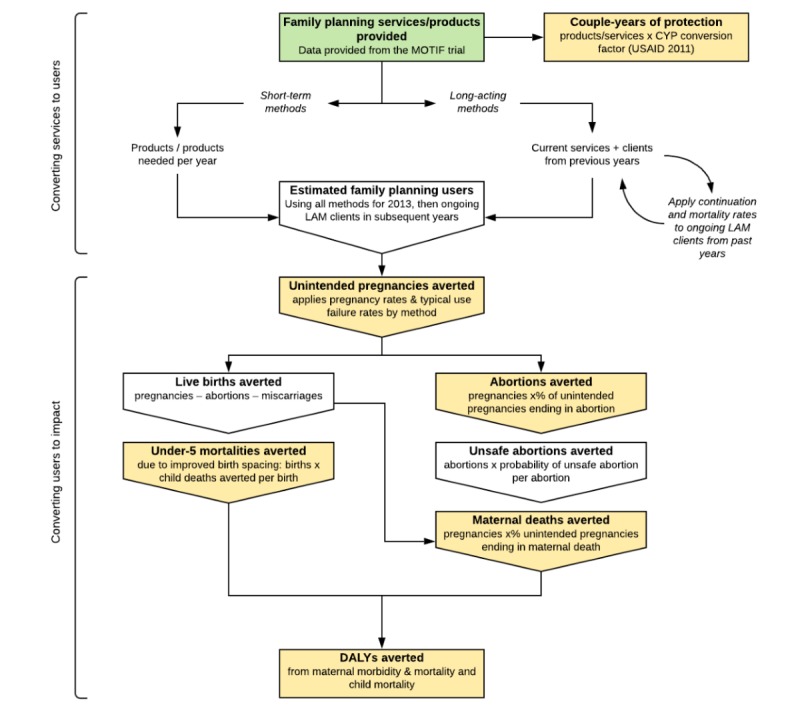
Marie Stopes International Impact2 model framework. Inputs, outputs, and processes used in the Impact2 model are illustrated, as they apply to this study. Green: inputs to the model from the MOTIF trial. Yellow: effects reported in this study. Adapted from Weinberger et al [21]. CYPs: couple-years of protection; DALYs: disability-adjusted life-years; LAM: long-acting method; MOTIF: MObile Technology for Improved Family planning.

Effects were estimated using the Marie Stopes Impact2 (version 4) modelling tool (illustrated in Figure 2) using the default settings in “organization” mode for Cambodia in 2013. This tool uses user-provided rates of contraceptive use, which we derived from the service provision model, to estimate effects such as disability-adjusted life-years (DALYs, calculated using 2010 Global Burden of Disease estimates [18]) averted and couple-years of protection (CYPs), taking account of effective usage, discontinuation, and failure rates and wastage for each contraceptive method, as well as country-specific rates for unintended pregnancies, induced abortions, maternal mortality, and under-5 mortality. Consistent with the Global Burden of Disease Methodology [18], no discounting of effects was applied and age weighting was uniform. Hutterite fertility rates account for the age structure of the population, which we adjusted in the model to reflect the MOTIF sample population. Discounting of fertility over the time horizon was also applied according to Hutterite rates. Effects were calculated to include the service lifespan of each contraceptive method (in the Impact2 model, we selected 10 years for IUDs and four years for implants). The Impact2 model includes assumptions based on published research. Of relevance to this study, 31% of pregnancies worldwide are unintended, and in Asia, 57% of unintended pregnancies end in abortion [2,19]. The full methodology and assumptions of the Impact2 model are described elsewhere [20,21].

### Costs

The base case analysis was performed from a societal perspective. Costs were collected in 2014 in US dollars (commonly used in Cambodia) and were expressed in constant 2011 purchasing power parity-adjusted US dollars.

For MSIC clinics, provider costs included medical consumables, personnel, and estimates of the time taken to provide each service. To account for overheads, 20% was added to personnel costs. For non-MSIC clinics, costs for personnel and overheads were not available, and commodity costs were assumed to be the same as those at MSIC clinics. Costs attributable to the intervention included airtime to deliver the mobile phone–based intervention and a proportion of fixed costs (computers and phones). MSIC personnel costs for training and delivery of the intervention were estimated from hourly wages and time spent on the intervention.

User costs included direct medical costs (service fees), direct nonmedical costs, and indirect costs of attending postabortion family planning services for the proportion of women who attended a separate appointment after their initial abortion. The average home-clinic round trip distance was multiplied by the per kilometer average price of motorcycle transport to obtain transport costs. If the client visited a different clinic, the estimated distance was reduced by one-third. Indirect costs to users were attributed to all women irrespective of formal employment status [22]. The time required for users to access each service was the sum of clinical time (reported by MSIC staff) and estimated travel time. Indirect costs attributable to repeat abortions were also included. In Cambodia, injectable and oral contraceptives are widely available at pharmacies, and therefore, costs were assumed to be negligible for clients obtaining these products from non-MSIC clinics. Unit costs and their sources are reported in Table 2 [22-24].

**Table 2:** Unit costs.

			Base	Deterministic range^a^	Probabilistic distribution	Comment/source
**Provider costs^b^**						
	**MOTIF^c^ intervention costs (per participant)**					
		Airtime: voice messages	0.79	0.39-1.18	Gamma	Actual costs from the MOTIF study
		Airtime: outgoing phone calls	2.16	1.08-3.25	Gamma	Actual costs from the MOTIF study
		Computer	1.34	0.67-2.01	Gamma	Actual costs from the MOTIF study
		Phone	0.2	0.1-0.3	Gamma	Actual costs from the MOTIF study
	**Family planning service commodities**					
		Oral contraceptive pill (one cycle)	0.29	0.15-0.44	Gamma	Direct cost reported by an MSIC^e^ clinic
		IUD^d^	0.4	0.2-0.6	Gamma	Direct cost reported by an MSIC clinic
		Medical abortion (Mariprist)	0.7	0.35-1.05	Gamma	Direct cost reported by an MSIC clinic
		Surgical abortion	5	2.5-7.5	Gamma	Personal communication with MOTIF trial authors
		Injectable contraceptive (one dose)	0.5	0.25-0.75	Gamma	Direct cost reported by an MSIC clinic
		Implanted subdermal contraceptive (Femplant)	8	4-12	Gamma	Direct cost reported by an MSIC clinic
		Long-acting contraceptive device removal	3	1.5-4.5	Gamma	Personal communication with MOTIF trial authors
	**Personnel (hourly)**					
		Midwife/health care service provider	2.36	1.18-3.54	Gamma	Direct cost reported by an MSIC clinic
		Counsellor	2.52	1.26-3.78	Gamma	Direct cost reported by an MSIC clinic
**User costs^a^**						
	**Direct costs**					
		IUD^d^ insertion	5	2.5-7.5	Gamma	Direct price to users reported by an MSIC clinic
		Implant insertion	25	12.5-37.5	Gamma	Direct price to users reported by an MSIC clinic
		Injectable (MSIC clinic)	1	0.5-1.5	Gamma	Direct price to users reported by an MSIC clinic
		Injectable (pharmacy)	0.73	0.37-1.1	Gamma	Direct price to users reported by a local pharmacy
		Oral contraceptive pill (MSIC clinic)	0.4	0.2-0.6	Gamma	Direct price to users reported by an MSIC clinic
		Oral contraceptive pill (pharmacy)	0.37	0.19-0.56	Gamma	Direct price to users reported by a local pharmacy
		IUD removal	2	1-3	Gamma	Direct price to users reported by an MSIC clinic
		Implant removal	3.75	1.8-5.63	Gamma	Direct price to users reported by an MSIC clinic
		Repeat abortion (surgical)	25	12.5-37.5	Gamma	Direct price to users reported by an MSIC clinic
		Repeat abortion (medical)	20	10-30	Gamma	Direct price to users reported by an MSIC clinic
	**Mobile phone**					
		Airtime to call a clinic/hotline (per min)	0.07	0.04-0.11	Gamma	Advertised cross-network charge in Cambodia
	**Transport**					
		Motorbike travel (per km)	0.22	0.11-0.33	Gamma	Data from Rozemuller et al [23]
		Average distance from clinic to home (km)	38.2	30.1-46.3^f^	Gamma	Data from the MOTIF study
	**Indirect costs**					
		Gross national income per capita	2534	2280.6-2787.4^g^	Gamma	World Bank development data [22]
		Gross daily income per capita	6.9	6.2-7.6^g^	Gamma	World Bank development data [22]
		Repeat abortion (total household indirect cost)	5.07	2.54-7.61	Gamma	Data from Potdar et al [24]

^a^The range used for deterministic analysis was 50% above and below the base case estimate unless otherwise indicated. This range was then assumed to represent the 95% confidence interval of the distribution indicated for probabilistic sensitivity analysis.

^b^Unit costs were combined to calculate the service level costs used in the model.

^c^MOTIF: MObile Technology for Improved Family planning.

^d^IUD: intrauterine device.

^e^MSIC: Marie Stopes International Cambodia.

^f^Range used is the 95% confidence interval from MOTIF data.

^g^Range used is 10% above and below the base case estimate.

### Cost-Effectiveness

Incremental cost and utility per 1000 participants were calculated by subtracting the estimated cost and each of the measures of effect (CYPs, pregnancies averted, abortions averted, under-5 mortality, maternal mortality, and DALYs) in the MOTIF intervention arm from those in the standard care arm. The incremental cost-effectiveness ratio (ICER) for each measure of effect was calculated by dividing incremental cost by incremental effect.

### Sensitivity Analysis

To estimate the effect of uncertainty, the model was subjected to deterministic and probabilistic sensitivity analyses [25]. Upper and lower range values were determined for each input parameter. Where possible, 95% CIs were derived from MOTIF trial data. Else, range values were calculated as 50% above and below the base case estimate to allow a wide range of uncertainty. The appropriate prior distribution for each parameter was chosen according to 2012 International Society for Pharmacoeconomics and Outcomes Research-Society for Medical Decision Making recommendations, and upper and lower range values were taken as the 95% CI of that distribution [26,27]. We assumed that changes in fees charged to users to access health services would not affect demand for those health services (ie, the price elasticity of demand for the services involved in the MOTIF intervention was zero).

The probabilistic sensitivity analysis consisted of a Monte-Carlo simulation with 1000 iterations randomized according to the probability distribution of each parameter. Contraceptive use outcomes for each iteration were inputted to the Impact2 model to produce the joint probability distribution for effects. Uncertainty introduced through the Impact2 model itself was not included, because information about parameters used in the Impact2 model was not available. Simulation results for ICERs assessed using CYPs and DALYs were plotted on the cost-effectiveness plane, and the cumulative probability for cost-effectiveness across a range of cost-effectiveness thresholds was visualized as a cost-effectiveness acceptability curve (CEAC) [28].

To understand the relevance of the cost-effectiveness analysis to decision makers, the results of the base case and sensitivity analyses were compared with the likely range of cost-effectiveness thresholds. Ochalek et al have described a method for empirically deriving cost-effectiveness thresholds in low- and middle-income countries, along with their estimate for a list of countries. The estimated cost-effectiveness threshold for Cambodia using this method ranged from US $58 to US $176 or 12%–35% of the gross domestic product per capita [29].

### Scenario Analysis

To understand the health financing implications of reducing or removing user fees, two scenario analyses were conducted to model the effect on costs from user and provider perspectives. Because user fees represent a transfer from users to providers, from a societal perspective, the net direct effect on costs is zero. For users, we calculated the average estimated cost per client in each scenario. For providers interested in the effect of user fees on cost-effectiveness, we calculated the estimated ICER from the provider perspective.

## Results

The incremental cost of mobile phone–based support from a societal perspective over a 12-month period was an additional US $8160.49 per 1000 clients, and it is reported along with costs to providers and users in Table 3. We estimate that an additional 518 CYPs are gained per 1000 clients receiving the MOTIF intervention and that this would avert 180 pregnancies, 103 abortions, and 99 DALYs. The ICER was US $82.57 per DALY averted and US $15.75 per additional CYP (Table 4). The ICER for DALYs averted fell within the cost-effectiveness threshold range.

**Table 3:** Base case cost and effect results for the MOTIF (MObile Technology for Improved Family planning) intervention versus standard of care.

		Intervention	Standard care	Incremental value
**Costs^a^ (US $)**				
	Provider	4079.74	−1625.20	5704.94
	User	15,906.83	13,451.28	2455.55
	Total	19,986.56	11,826.07	8160.49
**Effects^a^**				
	Couple-years of protection	1350.6	832.6	518.0
	Pregnancies averted^b^	441	260	180
	Abortions averted^b^	251	148	103
	U5^c^ mortalities averted^b^	3	2	1
	Maternal mortalities averted^b^	0	0	0
	DALYs^d^ averted	241.6	142.8	98.8

^a^Costs and effects are calculated per 1000 users.

^b^Rounded to the nearest whole.

^c^U5: under five.

^d^DALYs: disability-adjusted life-years.

**Table 4:** Base case incremental cost-effectiveness ratio (ICER) results for the MOTIF (MObile Technology for Improved Family planning) intervention.

Effect	ICER (US $ per unit of effect)
Couple-years of protection	15.75
Pregnancies averted	45.22
Abortions averted	79.33
U5^a^ mortalities averted	7659.96
Maternal mortalities averted	—^b^
DALYs^c^ averted	82.57

^a^U5: under five.

^b^No maternal mortalities were estimated to have been averted in either arm; therefore, no ICER calculation is possible.

^c^DALYs: disability-adjusted life-years.

**Figure 3:**
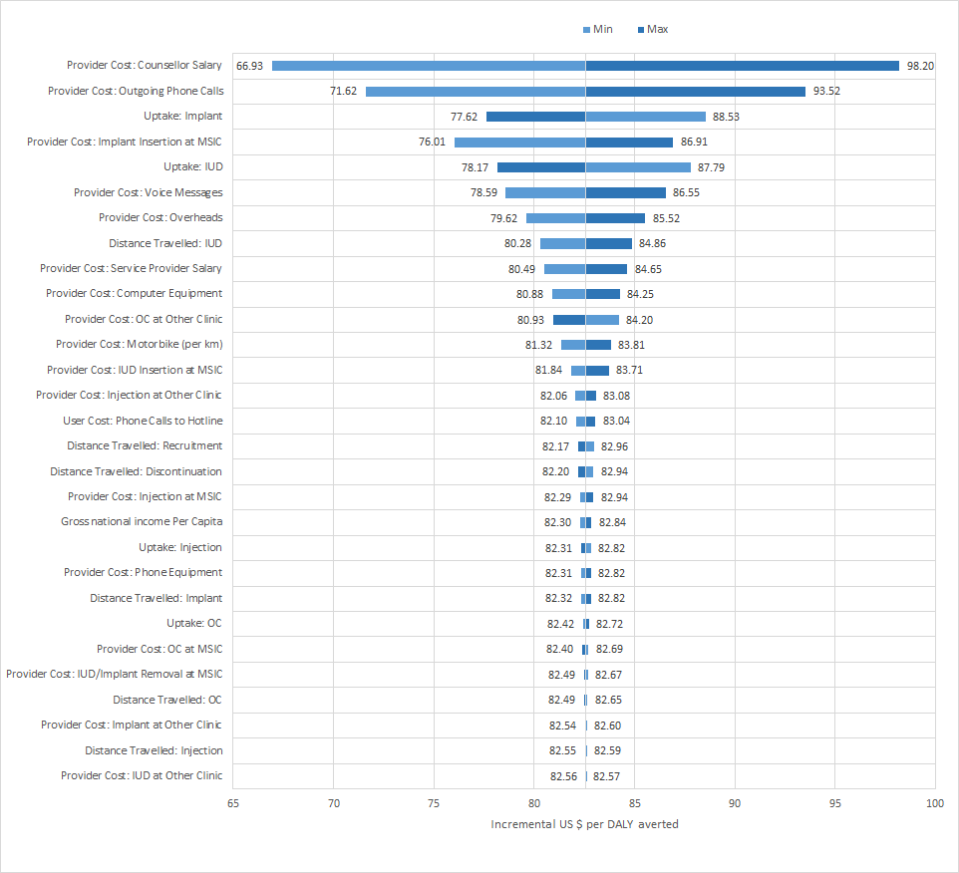
Tornado plot of deterministic sensitivity analysis using MOTIF intervention model parameters. For each parameter, the ICER was recalculated taking the upper and then lower deterministic range value. ICER ranges are centered on the ICER point estimate of US $82.57 per DALY averted. DALYs: disability-adjusted life-years; ICER: incremental cost-effectiveness ratio; IUD: intrauterine device; MOTIF: MObile Technology for Improved Family planning; MSIC: Marie Stopes International Cambodia; OC: oral contraceptive.

Results of the deterministic sensitivity analysis are presented as a tornado plot in Figure 3. The base case model was most sensitive to personnel costs, costs for phone calls and voice messages, the uptake and delivery of long-acting contraceptive methods (IUD or implant), and the percentage added to reflect overhead costs. For all parameters, the ICER estimated using upper and lower range values fell within the cost-effectiveness threshold range.

**Figure 4:**
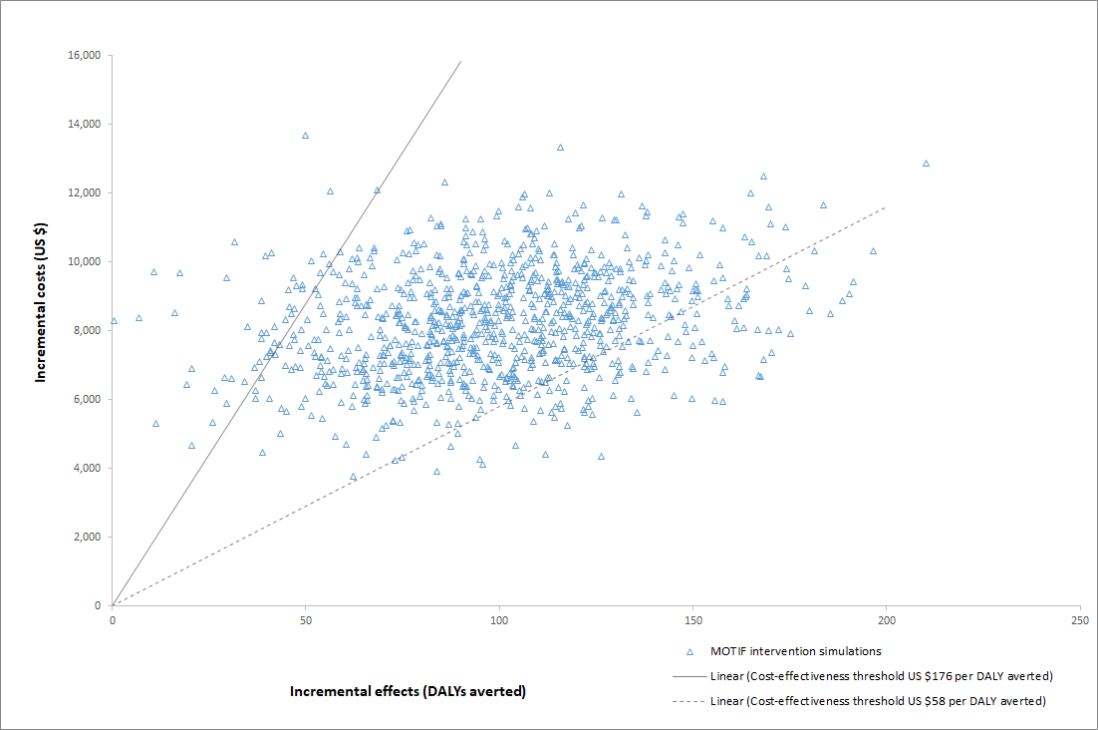
Monte-Carlo simulation results plotted on the cost-effectiveness plane, with effects measured in DALYs averted. Linear demarcations of the upper and lower bounds for the cost-effectiveness threshold for DALYs averted are included for comparison. DALYs: disability-adjusted life-years; MOTIF: MObile Technology for Improved Family planning.

**Figure 5:**
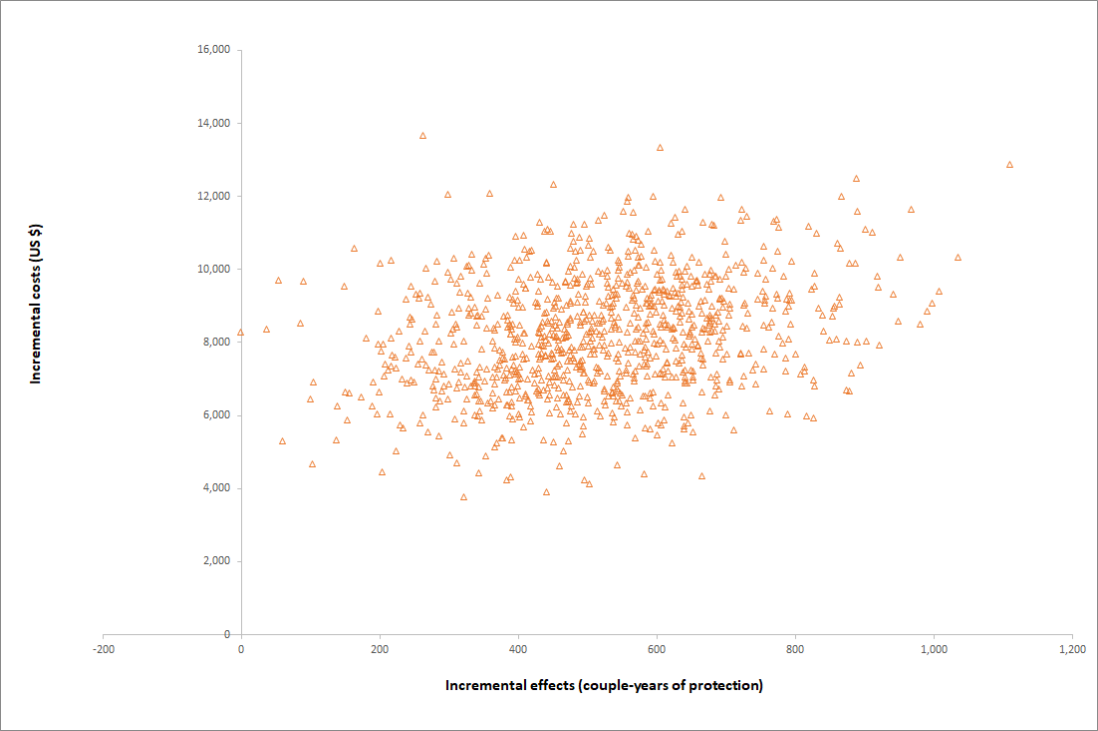
Monte-Carlo simulation results plotted on the cost-effectiveness plane, with effects measured in CYPs. CYPs: couple-years of protection.

**Figure 6:**
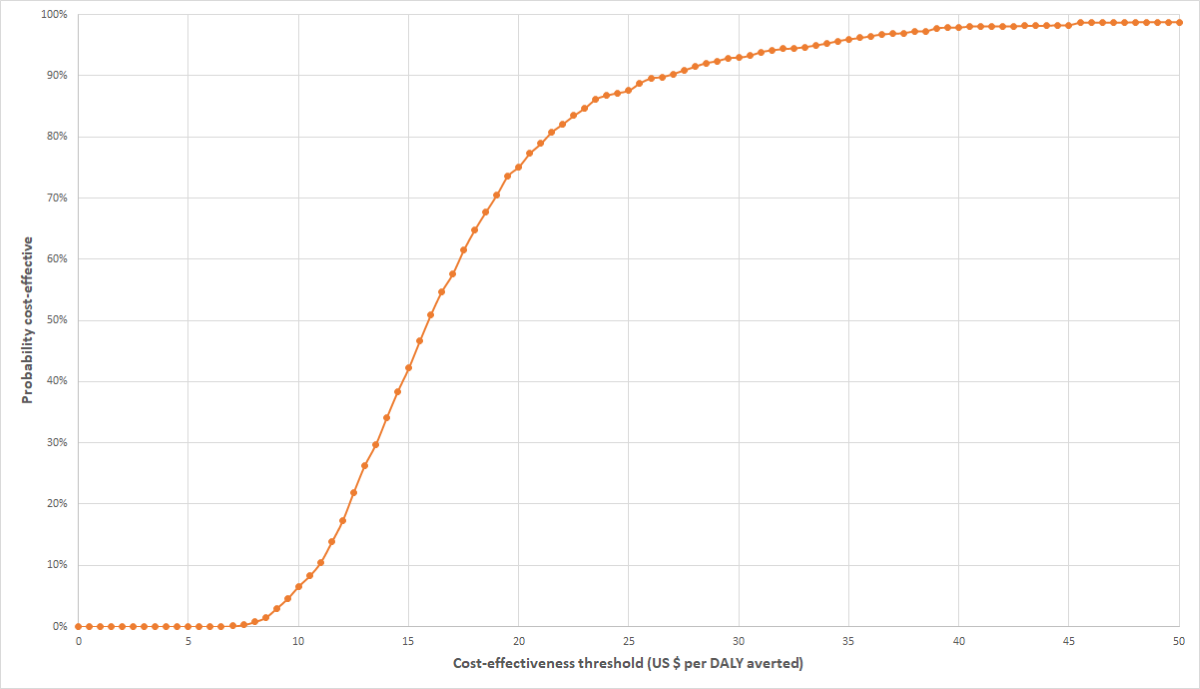
Cost-effectiveness acceptability curve derived from Monte-Carlo simulations of MOTIF intervention results, with effects measured in DALYs averted. DALYs: disability-adjusted life-years; MOTIF: MObile Technology for Improved Family planning.

**Figure 7:**
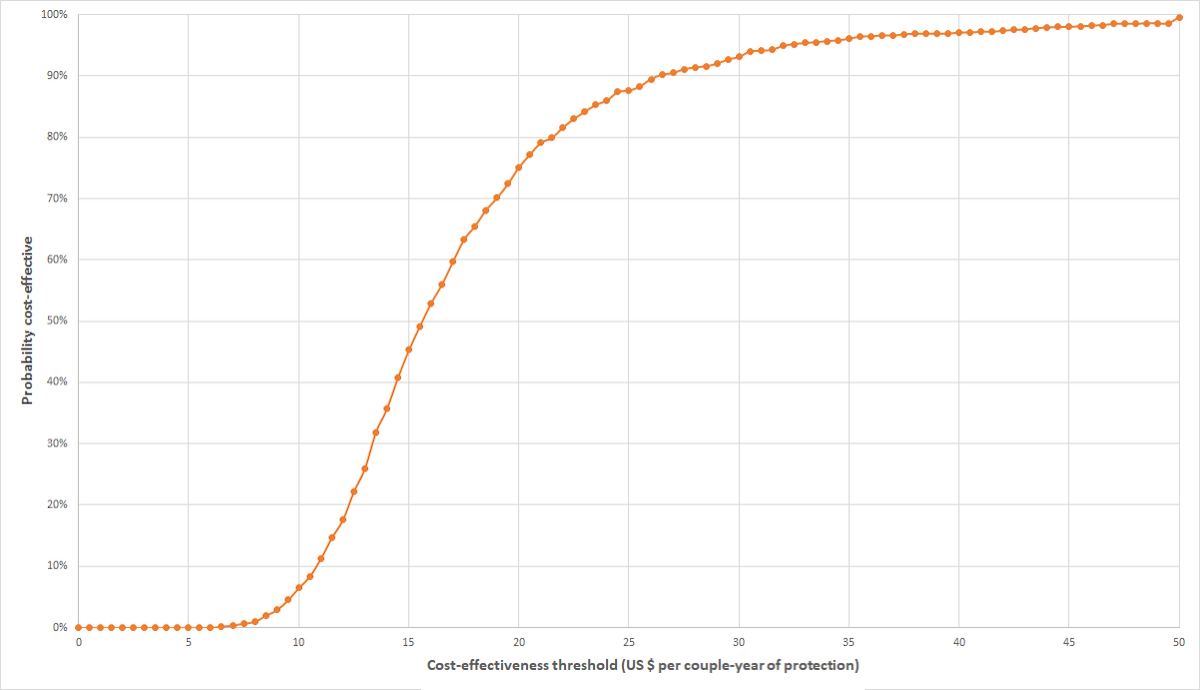
Cost-effectiveness acceptability curve derived from Monte-Carlo simulations of MOTIF intervention results, with effects measured in CYPs averted. CYPs: couple-years of protection; MOTIF: MObile Technology for Improved Family planning.

Simulations recorded for probabilistic sensitivity analysis are presented on the cost-effectiveness plane for DALYs averted and CYPs in Figures 4 and 5. Results for the two measures of effect appear very similar, albeit on a different horizontal scale, because the Impact2 modelling tool estimates approximately five times the number of CYPs achieved as DALYs averted, for any set of randomized inputs. On the plane for DALYs, most simulations lie within the cost-effectiveness threshold range. CEACs for the two measures of effect are shown in Figures 6 and 7. The CEAC measured per DALY averted shows that the intervention has an 11% probability of being cost-effective at the lower end of the cost-effectiveness range (US $58) and a 95% probability at the upper end of the range (US $176). A 50% probability of being cost-effective would be achieved at a cost-effectiveness threshold of US $83 per DALY averted and about US $16 per CYP.

Average costs from a user perspective and cost-effectiveness from a provider perspective, with either 50% or no user fees, are compared with costs and cost-effectiveness from a societal perspective in Table 5. With decreasing user fees, the average cost to users participating in the MOTIF intervention decreased from US $15,906.83 to US $8772.00 per 1000 participants (from 80% to 44% of the cost of the program from a societal perspective). The ICER from the provider’s perspective increased from US $57.72 to US $77.58 per DALY averted. With the removal of user fees and by considering the provider perspective, the MOTIF intervention remained within the range of cost-effectiveness thresholds for Cambodia.

**Table 5:** Costs for users and providers in scenarios involving variable user fees.

		Base case	Scenario 1 (50% user fees)	Scenario 2 (no user fees)	Societal perspective^a^
**Total cost per 1000 participants, user perspective (US $)^b^**					
	Intervention	15,906.83	12,339.41	8772.00	19,986.56
	Standard care	13,451.28^c^	10,864.97	8278.66	11,826.07
	Incremental	2455.55	1474.44	493.34	8160.49
**ICER^d^, provider perspective (US $ per unit of effect)^b^**					
	Couple-years of protection	11.01	12.91	14.80	15.75
	Pregnancies averted	31.61	37.05	42.49	45.22
	Abortions averted	55.46	65.00	74.54	79.33
	U5^e^ mortalities averted	5355.02	6275.95	7196.88	7659.96
	Maternal mortalities averted	—^f^	—	—	—
	DALYs^g^ averted	57.72	67.65	77.58	82.57

^a^Costs and ICERs from a societal perspective are included for reference. These results remain constant in each scenario, as the user fee represents a transfer from users to providers, but a net zero change from a societal perspective.

^b^Results are presented as total cost (direct and indirect) from a user perspective and ICER from a provider perspective to reflect the outcome of interest for the respective groups. Changes in demand resultant from the imposition of user fees have not been modelled as part of the scenario analysis.

^c^Under standard care with 100% user fees, the program provides income to providers.

^d^ICER: incremental cost-effectiveness ratio.

^e^U5: under five.

^f^No maternal mortalities were estimated to have been averted in either arm; therefore, no ICER calculation is possible.

^g^DALYs: disability-adjusted life-years.

## Discussion

### Principal Findings

This study demonstrates that the cost-effectiveness of the intervention delivered by mobile phone assessed in the MOTIF trial lies within the estimated range of the cost-effectiveness threshold for Cambodia. When assessing value in interventions to improve the uptake and adherence of family planning services, the use of interactive mobile phone messaging and counselling for women who have had an abortion should be considered as an option by policy makers. The MOTIF trial demonstrated that women randomized to an intervention delivered by a mobile phone were more likely to use long-acting contraceptive methods. Although these methods are known to be more cost-effective, the results of this study extend the evidence to show that an intervention delivered by a mobile phone favoring these methods is itself cost-effective.

### Strengths of the Study

This study has several strengths. Many of the cost and effect parameters are derived from trial and intervention delivery data rather than estimates from the literature, therefore improving the external validity of cost-effectiveness estimates within the Cambodian context. The use of the Impact2 model allows for replicable measurements of effects and comparison across studies. The cost-effectiveness estimates of the base case, the deterministic sensitivity analysis, and 96% of the probabilistic simulations fell within the chosen range of cost-effectiveness thresholds, and the threshold range was drawn from empirically derived cost-effectiveness threshold ranges, which are intended to realistically reflect what health systems are willing to pay [29,30]. We also included scenario analyses relating to the application of user fees for family planning services. Together, the design and results of this analysis might provide useful information when adapting the findings of the study to implementation, where affordability for public sector providers is likely to be an important factor.

Another strength of the study lies in the timely and important contribution to the literature linking innovations in mobile phone–based delivery with the delivery of family planning services. With the proliferation of mobile technology in the most rural and remote areas of the globe, there is great opportunity for harnessing mobile technology to reach women with life-saving health information. This study adds to the emerging body of knowledge about how to most effectively and efficiently achieve this aspect.

### Limitations of the Study

Deterministic testing indicated that estimated ICERs were particularly sensitive to counsellor personnel costs, estimated as a product of salary and time. However, these time estimates were not collected systematically, and they do not account for a run-in period of lower efficiency. Our estimates are therefore most relevant to a scaled-up intervention or a scenario where support by mobile phone is added to existing activities, for example, an established call center, where run-in time is reduced to a minimum. Process evaluations (unpublished) of the MOTIF trial intervention suggested that the link to a counsellor who could make an appointment if requested was a key to the success of the intervention, so programmatic implementation of a similar intervention should include these components. Although a component of training time was included in costs to deliver the intervention, the cost of ongoing technical support and training was not included. Sensitivity testing also indicated that the proportion of overheads attached to personnel costs produced a large change in ICER in comparison with other parameters. Overheads were not estimated as individual unit costs as part of the study, and thus, the approximation of overheads as a proportion of personnel costs could be improved.

Many of the cost parameters were estimated by personal communication with MOTIF study authors and staff, limiting the external validity of our results in other regions of Cambodia. Further, a range of 50% above and below the point estimate was used for sensitivity analyses. This was intended to capture a broad range of uncertainty in estimated costs, although it still may not accurately represent the cost of family planning services elsewhere in the country.

In the MOTIF trial, contraceptive use outcomes were self-reported by participants. Although this is the standard in family planning research, self-reported measures have been shown to overestimate contraceptive use and are susceptible to recall bias [31]. These outcomes were used as inputs for the Impact2 model, so the effects estimated using this model would be affected in a similar way to contraceptive use outcomes in the MOTIF trial.

The Impact2 modelling tool is based on a number of assumptions linking contraceptive service provision to health outcomes. Although these assumptions are founded in a strong evidence base, the evidence is drawn from the survey data of all women of reproductive age, and it is possible that patterns of contraceptive use and decision-making behavior might differ in a postabortion population. Although the model settings for Cambodia were used and the modelled population was adjusted to match the age distribution of participants in the MOTIF trial, it is possible that differences between the trial population and the population used to inform the Impact2 model, for example, the socioeconomic distribution, might result in errors. The authors of the Impact2 methodology note that estimates of under-5 mortality may be particularly unreliable owing to limited data on linkages among contraception use, birth spacing, and child mortality [20]. Despite these limitations, CYPs and DALYs are well-known measures of effect, and a focus on these outcomes increases the interpretability of this study in comparison with other interventions.

### Comparison With Existing Research

There is extensive related literature in the areas of mobile health (mHealth) and economic evaluation [32,33], of which, a number of studies relate specifically to family planning interventions. In a 2016 study by Mangone et al, a modelling approach was used to estimate the costs of a scaled-up mHealth intervention for reproductive health in Tanzania. This did not include a component of effectiveness; however, it did propose models for cost recovery based on mobile phone charges, in which costs were consistent with the findings of this analysis [34]. Zakiyah et al conducted a systematic review of economic evaluations of family planning interventions in low- and middle-income countries, identifying nine eligible studies, and in all of these, family planning interventions were found to be highly cost-effective [35]. There have been two systematic reviews of mobile phone–based interventions for family planning services; one focused on adults and the other focused on adolescents [9,10]. Both these reviews identified limited but promising evidence that mHealth interventions are effective for improving uptake and adherence to contraception, noting that there was sparse data from low- and middle-income countries. Our study is consistent with these findings and adds an important piece of economic evidence supporting the implementation of interventions delivered by mobile phone for family planning in these settings.

### Implications for Future Research and Health Policy

With the proliferation of cheap and accessible mobile phones and network access, even in rural and remote locations, there is substantial interest in taking advantage of mobile innovations to aid the delivery of family planning programs. The MOTIF trial intervention, which was recently included as a digital high-impact practice in family planning behavior change, is an example of a scalable mobile innovation [36]. However, it is difficult to make a case for scaled-up digital health interventions without an assessment of cost-effectiveness. This study demonstrates that the cost-effectiveness of the intervention in the MOTIF trial lies within the range of the cost-effectiveness threshold for Cambodia, thus supporting decision makers to include mHealth interventions in future family planning policies in Cambodia.

The sensitivity and scenario analyses included in this study provide useful details for health policy makers. Personnel costs and mobile phone costs have the greatest effects on the cost-effectiveness of the intervention and provide a useful focus for the business case that would accompany a scaled-up mHealth intervention. The cost and effect parameters used in this analysis were collected in a trial environment, whereas modest economies of scale could be achieved with wider implementation, for example, through automation of some call center tasks and bulk pricing agreements with network operators. From a user perspective, removal of user fees for services almost halved the average cost per participant in the intervention group. The effect of user fees on participation in family planning services was assumed to be zero in this study. Although there is likely to be some effect in practice, evidence from low- and middle-income countries suggests that contraceptive services are inelastic with respect to price [37]. These areas of uncertainty and opportunity are all fruitful areas for further economic and operational research.

Although this study provides useful evidence to support the cost-effectiveness of the MOTIF intervention, research to test and compare the cost-effectiveness of other interventions for improving the uptake of postabortion family planning services would improve the generalizability of this study to other settings.

### Conclusion

This study demonstrates that the use of an intervention delivered by a mobile phone to provide postabortion family planning counselling was cost-effective for increasing CYPs and for preventing pregnancy and abortion. It also provides a basis for further research on how this emerging technology can improve access to family planning services.

## Appendix

Multimedia Appendix 1 Detailed description of the MOTIF intervention.

## Abbreviations

CEAC: cost-effectiveness acceptability curve
CYPs: couple-years of protection
DALYs: disability-adjusted life-years
ICER: incremental cost-effectiveness ratio
IUD: intrauterine device
MOTIF: MObile Technology for Improved Family planning
MSIC: Marie Stopes International Cambodia
