# Supplementary material for: A Mobile Phone–Based Support Intervention to Increase Use of Postabortion Family Planning in Cambodia: Cost-Effectiveness Evaluation
Source: JMIR Mhealth Uhealth. 2020 Feb 25;8(2):e16276. doi: 10.2196/16276 (PMC7064963; doi:10.2196/16276)
Supplement: Multimedia Appendix 1 [file mhealth_v8i2e16276_app1.docx]

Appendix 1. Detailed description of the MOTIF intervention.

The conceptual framework for the intervention used in the MObile Technology for Improved Family Planning (MOTIF) study was based on literature reports on the determinants of contraceptive use and on links between contraceptive use and fertility.18 The intervention comprised six automated voice messages sent to participants’ mobile phones, at the time of their preference, during the 3 months following an abortion. Participants received the first message within 1 week of using abortion services and every 2 weeks thereafter. The message, recorded in the Khmer language, was as follows:

*Hello, this is a voice message from a Marie Stopes counsellor. I hope you are doing fine. Contraceptive methods are an effective and safe way to prevent an unplanned pregnancy. I am waiting to provide free and confidential contraceptive support to you. Press 1 if you would like me to call you back to discuss contraception. Press 2 if you are comfortable with using contraception and you do not need me to call you back this time. Press 3 if you would prefer not to receive any more messages.*

Participants who pressed 1 or who did not respond received a phone call from a counsellor. The phone calls were intended to encourage contraceptive use by increasing the client’s capability of using contraception by: (i) providing individualized information on a range of contraceptive methods; (ii) increasing the participant’s opportunity to use contraception, for example, by informing her where she could access specific methods near her residence; and (iii) increasing motivation by reinforcing knowledge of the benefits of contraception. At the participant’s request, the counsellor would also discuss contraception with her husband or partner.

Participants were also able to call the service and ask to speak to a counsellor. Those who chose to receive an oral or injectable contraceptive could opt to receive additional reminder messages appropriate to their method (e.g. on when to start a new packet of pills or when to receive a new injection). The sixth and final voice message was similar but also reminded the participant that this was the last message they would receive.

The intervention was delivered by trained counsellors at Marie Stopes International Cambodia. Voice messages were scheduled and sent using the open-source software program Verboice (InSTEDD, Palo Alto, United States of America). The cost of outgoing communications from the provider to the participant was met by Marie Stopes International Cambodia and the cost of calling into the service (i.e. a local call) was incurred by participants.
